# Identification of a Second Raccoon-Associated Polyomavirus

**DOI:** 10.1128/genomeA.00548-17

**Published:** 2017-06-29

**Authors:** Eileen M. Geoghegan, Nicole L. Welch, Michael J. Yabsley, Molly E. Church, Patricia A. Pesavento, Christopher B. Buck

**Affiliations:** a Lab of Cellular Oncology, CCR, NCI, NIH, Bethesda, Maryland, USA; b Southeastern Cooperative Wildlife Disease Study, Department of Population Health, College of Veterinary Medicine, and Warnell School of Forestry and Natural Resources, University of Georgia, Athens, Georgia, USA; c School of Veterinary Medicine, University of Pennsylvania, Philadelphia, Pennsylvania, USA; d Department of Pathology, UC Davis School of Veterinary Medicine, Davis, California, USA

## Abstract

Raccoon polyomavirus 1 (RacPyV1) is the suspected cause of an outbreak of fatal brain tumors among raccoons (*Procyon lotor*) in the western United States. Spleen samples from Georgia raccoons were screened for polyomaviruses. Although RacPyV1 was not detected, a previously unknown polyomavirus, which we designate RacPyV2, was identified and sequenced.

## GENOME ANNOUNCEMENT

Polyomaviruses are a family of nonenveloped circular DNA viruses believed to have coevolved with a diverse range of host animal lineages (1). Humans typically harbor chronic coinfections with multiple polyomavirus species. Other mammalian hosts also chronically harbor their own distinct homologs of various human polyomavirus clades. Although polyomavirus infections are not generally known to be associated with obvious symptoms in healthy individuals, human polyomaviruses can cause kidney disease, brain damage, and skin cancer in immunosuppressed individuals.

Investigation into cases of lethal brain tumors among wild raccoons (*Procyon lotor*) in California, Oregon, and Washington revealed that the tumors are stably infected with raccoon polyomavirus 1 (RacPyV1) (2, 3). Serologic testing indicates that wild raccoons in the eastern United States have antibodies that bind the VP1 major capsid protein of RacPyV1 (4). We attempted to isolate and sequence the RacPyV1-like virus that presumably infects raccoons in Georgia.

Spleen samples were collected from five raccoons from southern Georgia (University of Georgia IACUC approval numbers A2014 10-018 and A2010 10-186) and one skin sample was collected from a raccoon in northern California (collected during a routine, submitted, standard necropsy at the Anatomic Pathology Service of the Veterinary Medical Teaching Hospital at UC Davis). The samples were subjected to virion purification, rolling circle amplification, and MiSeq (Illumina) deep-sequencing, as previously described (5). Although RacPyV1 was not detected in any of the samples, one spleen sample had two separate contigs of approximately 2 kb each, showing similarity either to polyomavirus large T antigen (LT) or VP1 genes. Multiple PCRs were performed and subjected to Sanger sequencing, allowing coassembly of the two contigs into the complete genomic sequence of a single previously unknown polyomavirus. The prototype sequence was derived from a single spleen sample. A very closely related variant sequence (C2344G, T2437C, ∆2462-2464, G5094A) was detected in the skin sample. At the nucleotide level, the complete polyomavirus genome shows only 48% nucleotide identity with RacPyV1. We suggest that the new virus be named raccoon-associated polyomavirus 2 (RacPyV2).

Since the VP1 proteins of RacPyV1 and RacPyV2 are only 56% similar, it seems unlikely that they would be serologically cross-reactive. For example, human BK polyomavirus and Merkel cell polyomavirus, which show roughly 60% similarity in VP1 protein sequences, are not serologically cross-reactive (6).

The nearest homolog of RacPyV2 in GenBank is Moluccan fruit bat polyomavirus 1 (BatPyV5a, GenBank accession no. NC_026768), with 54% nucleotide identity across the entire genome. Phylogenetic analyses using the sequences posted at the “PyVE” website https://home.ccr.cancer.gov/Lco/PyVE.asp indicate that the predicted VP1 and VP2 capsid proteins of RacPyV2 are divergent from other known polyomaviruses, showing only loose phylogenetic affiliation with Almi-clade species, such as BatPyV5a. The predicted LT protein of RacPyV2 shows distant but well-supported phylogenetic affinity with human polyomavirus 10 and Saint Louis polyomavirus, both of which have Almi-clade LT proteins (1). RacPyV2 could thus be described as a candidate carnivoran Almipolyomavirus. Consistent with this description, RacPyV2 encodes an alternative LT ORF (ALTO) and a potential middle T antigen (7).

### Accession number(s).

RacPyV2 has been assigned GenBank accession number KY549442.
